# Getting breastfeeding started under pandemic visiting restrictions: lessons learned in Germany

**DOI:** 10.1186/s13006-024-00664-7

**Published:** 2024-09-13

**Authors:** Mathilde Kersting, Erika Sievers, Nele Hockamp, Hermann Kalhoff, Thomas Lücke

**Affiliations:** 1 Research Department of Child Nutrition, University Hospital of Pediatrics and Adolescent Medicine, St. Josef-Hospital, Ruhr-University Bochum, Alexandrinenstraße 5, 44791 Bochum, Germany; 2 Haale, Germany; 3 Pediatric Clinic Dortmund, Dortmund, Germany

**Keywords:** Breastfeeding initiation, Breastfeeding support, Maternity ward staff, Hospital visiting restriction, COVID-19 pandemic, Germany, Cross-sectional study

## Abstract

**Background:**

The COVID-19 pandemic contact restrictions considerably changed maternal visiting contacts during the time in which breastfeeding is initiated. We wanted to know how maternity ward staff and mothers rated the conditions of starting breastfeeding under contact restrictions.

**Methods:**

In the Breastfeeding in North Rhine-Westphalia (SINA) study, Germany, 2021/22, chief physicians as well as ward staff from 41 (out of 131) maternity hospitals (82 members of the healthcare sector in total) were surveyed by telephone concerning structural and practical conditions for breastfeeding support before and during the pandemic; 192 (out of 426 eligible) mothers answered an online-questionnaire about their breastfeeding experiences at 2 weeks and 2 months after birth.

**Results:**

In almost all of the hospitals, visits were restricted due to the pandemic, with the exception of the primary support person. After more than one year of pandemic experience, the ward staff were convinced that the restrictions were mostly positive for the mothers (97.6%) and for the ward staff themselves (78.0%). A total of 80.5% of the ward staff would maintain the restrictions beyond the pandemic. The mothers themselves mostly rated the restrictions in the hospital as being just right; moreover, many mothers voluntarily maintained the restrictions at home, at least in part.

**Conclusions:**

The unprecedented visiting restrictions in hospitals during the pandemic were like an “experiment” born out of necessity. Restricting visiting arrangements may be an underestimated beneficial component for the development of the mother-infant dyad in perinatal breastfeeding care, particularly in healthcare systems where almost all births occur in the maternity hospital.

**Trial registration:**

German Clinical Trials Register (DRKS) (DRKS00027975).

## Background

The early postnatal period is important for the establishment of successful breastfeeding [1, 2]. In Germany, almost all births (98%) occur in hospitals providing maternity services [3]. There are currently approximately 600 maternity hospitals in Germany [4]. Approximately 100 hospitals (17%) are designated ‘Baby-friendly’ [5]. In the last 20 years, postpartum stay in the hospital has decreased from a median of 5.3 days in 1997/98 to 3.0 days in 2017-19, as reported in the national surveys on “breastfeeding and infant nutrition in Germany”, which are known as SuSe I and SuSe II [6].

Before the COVID-19 pandemic, generous regulations on the number and duration of visitors were common in German hospitals. COVID-19 led to strict short-term contact restrictions beginning in April 2020. Hospitals had to comply with nationwide and state-specific regulations, such as in North Rhine-Westphalia (NRW), and were basically free to regulate visits themselves within this framework [7]. In maternity hospitals, visitors from outside the hospital were generally no longer allowed, but exceptions were permitted for the primary support person [7]. Considering the physiological course of breastfeeding initiation and the psychological course of parent-child interaction it seems obvious that such fundamental interventions in maternal social contacts could have an impact on breastfeeding.

In the first year of the pandemic, 6,201 research articles were published on COVID-19 and ‘maternal and child health, and nutrition’, including 445 articles on breastfeeding [8]. Most of them dealt with infection issues [8]. Later, studies more often addressed risks for maternal mental health and wellbeing [9, 10]. Surprisingly, the impact of perinatal contact restrictions on breastfeeding have been rarely addressed [10, 11], and when it is addressed, it is mostly limited to the special case of the support person being present at birth [12].

The 2021/22 SINA (Breastfeeding in North Rhine-Westphalia) study examined hospital breastfeeding management and maternal breastfeeding behaviors during the COVID-19 pandemic in the federal state of North Rhine-Westphalia, Germany [13]. Using the COVID-19 pandemic as an “experiment” born out of necessity, the aim of this data analysis was to explore how maternity ward staff and mothers rated the conditions for starting breastfeeding under contact restrictions.

## Methods

### Overview

The SINA study was performed in NRW, which is the most populated of the 16 federal states in Germany. The study comprised two parts: a state-wide cross-sectional quantitative survey of maternity hospitals focusing on perinatal breastfeeding management and experiences before and during the pandemic (‘hospital study’) and a regional prospective survey of mothers during the first 2 months after birth focusing on breastfeeding practices and pandemic experiences (‘mother study’) [13]. The data were collected between October 2021 and March 2022, i.e., during a period in which hospitals had experienced the pandemic for more than one year. No major changes to official COVID-19 hospital visit regulations occurred during the data collection period [14].

The study design and the data assessments in SINA were based on the nationwide SuSe II study 2017-19 [6]. Obligatory for participation was the written informed consent of hospitals and mothers. The SINA study was approved by the Ethics Committee of the Medical Faculty of the Ruhr University Bochum (Reg-Nr. 21-7322, 28.08.2021) and registered at German Clinical Trials Register (DRKS) (DRKS00027975).

### Hospital study

#### Recruitment

All maternity hospitals in NRW were eligible for participation. The heads of the gynecological departments were invited by postal letter. In the case of missing feedback, hospitals were reminded by phone calls and additionally reminded by fax and a video invitation from the person in charge of the study. Study participation included a telephone interview of the head of the department and separately of a person who was responsible for breastfeeding support on the maternity ward. In recognition, the ward was offered a gift worth 30 Euros.

Telephone interviews.

The main topics of the survey were agreed in advance with the heads of department of four large maternity hospitals in Bochum and Dortmund. The interview questionnaire for SINA was developed on the basis of the SuSe II study [6]. It included mostly closed questions and some open questions. Closed questions concerned various factors such as the percentage of Caesarean sections and skin-to-skin contact between mother and child soon after birth (yes/no). Open questions concerned various parameters such as potential consequences of the pandemic for pre- and postnatal breastfeeding information for mothers or the practice of supplemental feeding on the ward. The heads of department were mainly interviewed about structural aspects of breastfeeding management including the availability of a breastfeeding coordinator, the offer of staff training on breastfeeding support, and potential changes due to the pandemic. In total, 66 questions including sub-questions were asked. The interviews with ward staff mainly focused on practical issues of breastfeeding support for mothers, such as helping mothers to latch their baby and availability of breastfeeding aids on the ward. In total, 67 questions including sub-questions were asked in the questionnaire.

The interviews also addressed pre-pandemic routines and changes during the pandemic. Participants were also asked about their views on the impact of the pandemic on breastfeeding support. The interviews lasted about 45 min. The interviews with the heads of department were conducted by the person in charge of the study, and the interviews with the ward staff by the same study member. In addition to the interviewer, a second member was present and entered the answers into the data system. The interviews were recorded to enable subsequent validity checks.

### Mother study

#### Recruitment

Mothers were recruited from the four large abovementioned maternity hospitals in the Ruhr Area (Bochum, Dortmund), which is an industrial centre in NRW. Three of the four hospitals had a catchment area with predominantly low socioeconomic status. Mothers who were eligible for participation in the study were consecutively invited on the maternity ward by study personnel. The inclusion criteria were: a healthy, fullterm newborn (birthweight ≥ 2500 g, gestational age ≥ 37 weeks, no admittance to a neonatal intensive care unit), maternal age of at least 18 years, no maternal health problems, sufficient maternal knowledge of the German language, internet access, and an email address.

Study participation required participants to complete a web-based questionnaire 2 weeks and 2 months after birth. In recognition, mothers were offered a brochure from the Research Department of Child Nutrition (FKE) with recommendations for infant feeding.

#### Online questionnaires

The digital questionnaires were sent by email and administered by the Fraunhofer Institute for Software and System Technology ISST, Dortmund. The questions addressed the current nutrition of the infant at the time of interest such as at 2 weeks of age. All liquids that the child received were asked individually, e.g. “What does your child receive at present?” Based on this information, exclusive breastfeeding was defined by the study staff as: No liquids or solids other than breastmilk (except for prescribed medicines, oral rehydration solution, vitamins and minerals) as defined by the WHO [15] and the German National Breastfeeding Committee [16].

The 2-week questionnaire additionally asked retrospectively about the infant’s feeding status during hospital stay and at discharge. In addition, maternal characteristics were assessed at 2 weeks, variables such as breastfeeding problems, reasons for stopping breastfeeding, type and timing of breastfeeding information before and after birth were assessed as well. In both surveys, mothers were also asked about their experiences with breastfeeding support, especially during the pandemic. If the questionnaires were not answered within a predefined time frame, mothers received an email as a reminder, followed by a phone call. Questionnaires that were not returned on time led to the exclusion of the mother from further participation in the study.

### Data presentation

Responses from the hospital interviews were categorized into predefined categories (inductively) or via the grouping of free answers afterwards according to their meaning (deductively).

Descriptive data analysis was performed by using the IBM^®^ SPSS^®^ Statistics Version 25.0 software package for Windows 2016 (IBM Corp.). Percentages for categorical variables or frequencies for continuous variables were used for data presentation. To determine differences between categorical characteristics of mothers, the Fisher’s exact test or the Fisher-Freeman-Halton exact test of independence if the contingency table was larger than 2 × 2 was used. *P* - values < 0.05 (two-sided) were considered to indicate statistical significance.

## Results

### Hospitals

#### Hospital characteristics

Of the 135 invited maternity hospitals, four hospitals had to be excluded (currently no maternity services/hospital management, being merged with another hospital); of the 131 eligible hospitals, *n* = 41 (31%) agreed to participate and provided a full interview with the head of department and the person responsible for the ward.

The participating hospitals most often had an annual birth rate between 1000 and 1999 births and were located in a region with a medium socioeconomic background (Table 1). The proportion of hospitals with a Baby-friendly designation was greater than nationwide in Germany (29% vs. 17%, respectively). Mothers stayed in the hospital for (median) 2.5 days after vaginal birth and 3.5 days after Caesarean section. The recommendations for breastfeeding support in hospitals from WHO and UNICEF [17], which have been adapted to Germany [18], were met in a wide range, as shown by the examples of breastfeeding on demand (in 100% of hospitals) and early initiation of breastfeeding in Caesarean births (in 30% of hospitals).

**Table 1:** Hospital characteristics

Hospital structures and breastfeeding management	*n* = 41	%
Annual birth rate^a^		
< 1000	8	20
1000–1999	25	61
≥ 2000	8	20
Socioeconomic status of catchment area^b^		
High	8	20
Medium	23	56
Low	10	24
Perinatal level of care		
Maternity and neonatal pediatric hospital combined	20	50
Maternity and neonatal pediatric hospital separate	5	13
Maternity hospital only	15	38
Baby-friendly Hospital designation		
Yes	12	29
No	29	71
Maternal length of hospital stay pp (days)^c^		
Vaginal birth^d^	2.5 (2.0–3.0)	
Caesarean section^e^	3.5 (3.0–4.0)	
Fulfilment of breastfeeding promotion recommendations (WHO/UNICEF)		
Breastfeeding on demand	41	100
Practical support of mothers	33	81
Early initiation of breastfeeding (< 1 h after birth)		
Vaginal birth	18	44
Caesarean section	12	29

^a^In 2020. ^b^Extent of unemployment, households in basic security (Hartz-IV), single parents. ^c^Median and 25th–75th interquartile range. ^d^*n* = 40. ^e^*n* = 38

Deviations due to rounding

#### Visiting regulations

**Fig. 1:**
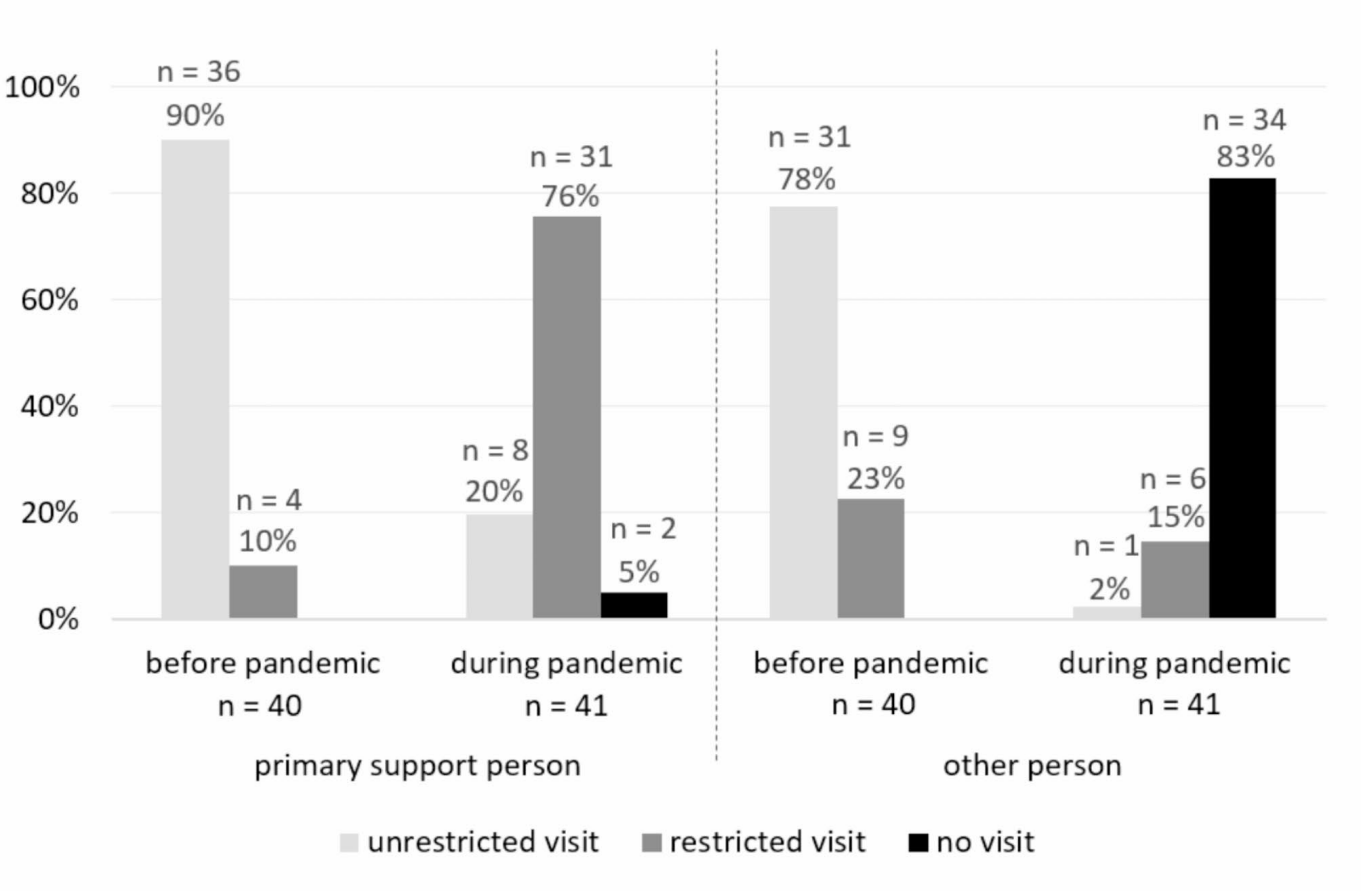
- Visiting regulations in hospitals before and during the pandemic

Before the pandemic, visits by the mother’s primary support person were practically unrestricted in almost all of the hospitals and in the vast majority also for other relatives or friends (other person). With the outbreak of the pandemic, the situation fundamentally changed: specifically, in almost all of the hospitals, the primary support person was still allowed to visit, but mostly only for a limited amount of time 76, whereas other visitors were completely excluded in the vast majority of hospitals (Fig. 1).

#### Perceived consequences

In 78% (32/41) of hospitals, the ward staff perceived positive effects of the visiting restrictions for themselves, for example, supporting mothers with infant feeding was found to be easier and the staff felt more satisfied. Almost all of the ward staff (98%, 40/41) perceived effects for mother and the newborn to be positive, specifically, mothers were able to concentrate on their baby and they felt relaxed.

Ward staff were also asked about their assessment of pre-pandemic breastfeeding rates at discharge. They estimated the proportion of any breastfeeding to be 85% on average (range: 60–99%) (out of *n* = 40) and the proportion of exclusive breastfeeding to be 67% on average (range: 20–98%) (out of *n* = 39). A total of 38% (15/40) of ward staff perceived an increase in breastfeeding at discharge during the pandemic.

From the ward staff perspective, 81% (33/41) considered it desirable to maintain the visiting restrictions after the pandemic, at least to a limited extent; for the others, this scenario was discussed (7%, 3/41) or there was no interest or competence to decide on a change in visiting practices (12%, 5/41).

### Mothers

#### Maternal characteristics

**Fig. 2:**
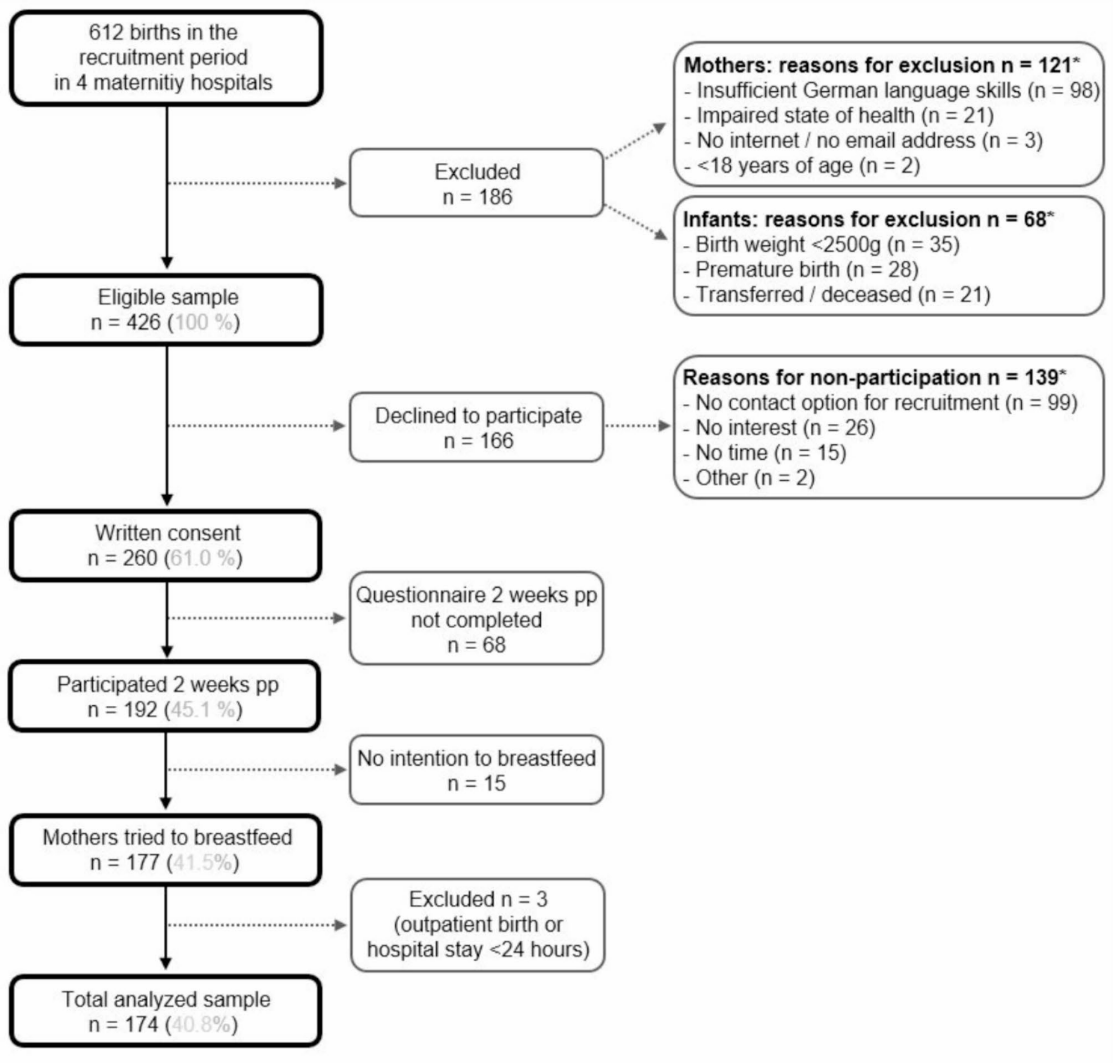
- Flow chart of recruiting and selecting the sample of mothers

Of the total 612 births in the four maternity hospitals during the recruitment period, 426 mother-infant-pairs met the inclusion criteria. Of the eligible mothers, 61.0% (*n* = 260) agreed to participate. Of those, 45.1% (*n* = 192) answered the first questionnaire 2 weeks postpartum and 42.3% (*n* = 180) also answered the second questionnaire 2 months postpartum, which corresponds to a follow-up of 93.8%. Finally, a total sample of 174 mothers having tried to breastfeed was analyzed here (Fig. 2).

Most of these mothers were aged between 30 and 34 years (mean: 32.2, range: 19–42 years), had a higher level of education, were primiparous, had a vaginal birth and stayed in the hospital for 2 or 3 days (Table 2, first column). The rate of exclusive breastfeeding at 2 weeks after birth was 71.8%. + multiple answers possible.

**Table 2:** Maternal characteristics and stratification of mothers according to their perception of the hospital visiting restrictions

	Total sample^a^		Perception of hospital visiting restrictions, *n* (%)				
Maternal characteristics			“Just right”		“Too strict”		
	*n* = 174	%	*n* = 116	66.7%	*n* = 58	33.3%	*p* - Value
Age (years)							
18–29	45	25.9	20	17.2	25	43.1	
30–34	80	46.0	60	51.7	20	34.5	**0.002**
35 – ≥ 40	49	28.2	36	31.0	13	22.4	
Level of education^b^							
Basic	11	6.4	7	6.1	5	8.6	
Secondary	31	18.0	17	14.8	14	24.1	0.234
Higher secondary	130	75.6	91	79.1	39	67.2	
Marital status^c^							
Married Single	130 42	75.6 24.4	92 22	80.7 19.3	38 20	65.5 34.5	**0.038**
Employed before maternity leave							
Yes No	132 42	75.9 24.1	96 20	82.8 17.2	36 22	62.1 37.9	**0.004**
Parity							
1st child	113	64.9	78	67.2	35	60.3	
2nd child	41	23.6	27	23.3	14	24.1	0.480
>= 3 children	20	11.5	11	9.5	9	15.5	
Birth							
Vaginal	112	64.4	77	66.4	35	60.3	
Unplanned Caesarean section	33	19.0	20	17.2	13	22.4	0.635
Unplanned Caesarean section	29	16.7	19	16.4	10	17.2	
Complications during birth							
Yes No	35 139	20.1 79.9	19 97	16.4 83.6	16 42	27.6 72.4	0.108
Close person present at birth							
From the start	142	81.6	94	81.0	48	82.8	
Partially	26	14.9	19	16.4	7	12.1	0.510
Not at all	6	3.4	3	2.6	3	5.2	
Length of hospital stay pp (days)							
≤ 1	10	5.7	5	4.3	5	8.6	
2	68	39.1	45	38.8	23	39.7	0.624
3	61	35.1	43	37.1	18	31.0	
> 3	35	20.1	23	19.8	12	20.7	
Room occupancy							
Single Multiple	120 54	69.0 31.0	88 28	75.9 24.1	32 26	55.2 44.8	**0.009**
Exclusive BF 2 weeks pp							
Yes No	125 49	71.8 28.2	86 30	74.1 25.9	39 19	67.2 32.8	0.374

The Fisher’s exact test or the Fisher-Freeman-Halton exact test of independence was performed for the categorical variables. *P*-values < 0.05 (two-sided) were considered to be significant. EBF, exclusive breastfeeding; pp, postpartum. ^a^*n* = 3 excluded from *n* = 177 (“Does not apply I, had an outpatient birth or left the hospital within one day” *n* = 2; visiting restrictions not strict enough *n* = 1). ^b^No answer: *n* = 1 / no school-leaving qualification *n* = 1 grouped with basic level of education. ^c^other *n* = 2

#### Perception of visiting regulations in hospitals

In the 2-week questionnaire, mothers were asked about their experiences with visiting restrictions since birth. More breastfeeding mothers (66.7%) felt that the visiting restrictions at the hospital were generally just right, whereas for a smaller proportion (33.3%) they were too strict. Stratification of mothers according to their perception of the visiting restrictions (Table 2) showed, that mothers for whom the restrictions were too strict were younger, often not married, less often employed before maternity leave, and more often had a shared room at hospital than mothers who perceived the visiting restrictions to be just right.

With regard to breastfeeding, 59.2% of mothers rated the visiting restrictions in the hospital as being positive: they had plenty of rest and reported that they were generally able to breastfeed well. For a smaller proportion (27.6%), the restrictions were only partially positive: mothers were able to breastfeed well but often felt alone, or they had plenty of rest but received less breastfeeding support. Explicit negative effects were less frequently reported (7.5%), mainly feeling lonely and insecure about breastfeeding. A small proportion of mothers (4.0%) did not feel any impact of the restrictions on breastfeeding.

At home, most breastfeeding mothers had continued to restrict visits (54.6%) or had relaxed the restrictions (17.8%), whereas others (27.6%) no longer restricted visits.

## Discussion

### Overview

This data analysis highlights a very specific consequence of the COVID-19 contact restrictions in the perinatal breastfeeding environment. After more than a year of pandemic experience, maternity ward staff clearly viewed the visiting restrictions as being positive for both, mothers and staff. The fact that staff reported having more time to support mothers may have made it easier for mothers to focus on breastfeeding.

The mothers’ views on the hospital visiting restrictions confirm the results from the hospitals: mothers experienced a calm hospital atmosphere and often felt that they could breastfeed well. These positive experiences in the hospital may also have motivated mothers to maintain visiting restrictions, at least partially, at home. Despite the more permissive contact opportunities at home, a catch-up effect of missed hospital visits does not appear to have occurred.

The desire of the ward staff to at least partially maintain the restrictions beyond the pandemic confirms their positive assessment of the restrictions on breastfeeding success.

Overall, some important aspects of our findings for hospitals and mothers are reflected in the pandemic experiences reported by lactation consultants in hospitals in the US [19]. They reported that babies tended to be poorly breastfed when visitors were around and mothers did not rest. In contrast, when no visitors were around, mothers felt comfortable with the baby and parents attempted to breastfeed for longer periods of time before asking for supplemental feeding to be introduced. At the same time, lactation consultants found it difficult to find a good compromise of visiting arrangements that would ensure both optimal professional breastfeeding support and the satisfaction of the families.

### Experience from other studies

The COVID-19 pandemic has led to various interventions that could be relevant for breastfeeding, thus making it difficult to compare research results. Clearly describable measures such as official contact restrictions have rarely been addressed in relation to breastfeeding [20]. In particular, the experiences of hospitals in limiting visits have not been adequately studied, although these policy factors of hospitals have a significant influence on the initiation of breastfeeding [1, 12].

Similar to our quantitative survey, the authors of a qualitative study in Spain reported that visiting restrictions tended to be positively perceived in relation to breastfeeding outcomes [21]. Mothers perceived contact restrictions as predominantly positive for breastfeeding and bonding with the child because disturbances from outside were eliminated. However, as in our study, feelings of loneliness and missing the family were also expressed [21].

The severity of hospital visit restrictions also seems to play a role, as two studies from Italy have suggested [11, 22]. In one hospital in which partners had no access, breastfeeding rates after birth and in the following three months were lower than those reported several years earlier [22]. In another hospital where the presence of the partner was only partially restricted, breastfeeding rates at discharge were not affected, although mothers felt more anxious and less supported by hospital staff [11].

In a nationwide online survey of maternity hospitals in the US in 2020, hospital self-assessed breastfeeding rates at discharge remained approximately the same in 68.9% of hospitals and increased in 11.3% of hospitals since the outbreak of the pandemic [23]. Although this is only an estimation, the ward staff of our hospitals were more optimistic (37.5%) that breastfeeding rates at discharge increased during the pandemic.

### Lessons learned

#### Forced experiment

The COVID-19 pandemic, with its unprecedented and stringent visiting restrictions in hospitals is akin to an ‘experiment’ born out of necessity, wherein there were interferences with the social life of patients and the daily work of medical and care staff. Such clearly defined and abrupt interventions in the breastfeeding environment would neither be feasible as a formal research study, nor ethically acceptable under Western perinatal conditions. Therefore, the experience with the pandemic could help to identify favorable and unfavorable social conditions for a smooth breastfeeding initiation. Moreover, our ‘mother study’ suggests that younger, single and non-employed women might be target groups for specific research on the influence of social factors in the perinatal situation on maternal wellbeing.

#### Short postnatal hospital stay

In the SINA study, the hospital stay after a vaginal birth was 2.5 days before the pandemic and was estimated to be even shorter by 60% of hospitals during the pandemic [13]. In the US, the vast majority of hospitals (72.9%) have shortened their hospital stays to less than 48 h due to the pandemic [23], which is a duration defined as ‘shortened stay’ [24] and which requires increased breastfeeding support.

With approximately 50% of mothers in Germany reporting breastfeeding problems in the first two weeks postpartum [13, 25], the need to improve early breastfeeding support is evident. Breastfeeding problems have been associated with a shortened duration of exclusive breastfeeding [26]. It would be interesting to have data on whether effective perinatal breastfeeding support would be enhanced by limitation of visitors in the immediate postpartum period and whether avoidable disturbances of the maternal-child interaction would be less frequent, and could help to prevent the development of problems around breastfeeding.

### Strengths and weaknesses

One of the strengths of the study is the transferability of the findings to countries with similar social and healthcare systems. Although the rather low participation rates of hospitals and mothers may have favored an overrepresentation of breastfeeding friendliness, the pandemic restrictions equally applied to all hospitals and mothers, thus the consequences are likely to be transferable.

A further limitation of the data is that the changes in breastfeeding rates perceived by maternity ward staff during the pandemic could not be verified because a control situation was not feasible. Data reported by hospital staff may have resulted in social desirability bias. Confirmation of the findings in further studies is needed.

### Future prospects

Visiting arrangements may be one of the underestimated socioemotional components of breastfeeding support, as they may have an impact on maternal wellbeing thus affecting the probability of successful breastfeeding initiation.

Further studies are needed to distinguish the specific role of postnatal visiting regulations from other factors of breastfeeding support. At present, it can be suggested that visiting regulations in the maternity ward should combine the clinical and social needs of the young family. Practically, this could mean that visiting arrangements in the maternity ward should be reasonably channeled, even if this may seem surprising in a liberal social environment. In this way, new perspectives for postpartum breastfeeding support could be developed from the pandemic experience.

## Data Availability

The datasets used and analysed during the current study are available from the corresponding author on reasonable request.

## Abbreviations

FKE: Research Department of Child Nutrition
NRW: North Rhine-Westphalia
SINA: Breastfeeding in North Rhine-Westphalia
SuSe: Breastfeeding and infant nutrition in Germany
